# Correlation of Serum Albumin Level to Lung Ultrasound Score and Its Role as Predictors of Outcome in Acute Respiratory Distress Syndrome Patients: A Prospective Observational Study

**DOI:** 10.1155/2021/4594790

**Published:** 2021-12-07

**Authors:** Souvik Chaudhuri, Sagar S. Maddani, Shwethapriya Rao, Sirish Gauni, N. R. Arjun, Pratibha Todur, Nitin Gupta

**Affiliations:** ^1^Department of Critical Care Medicine, Kasturba Medical College, Manipal Academy of Higher Education, Manipal 576104, Karnataka, India; ^2^Department of Adult Critical Care Medicine, St. George's University Hospital, NHS Trust, London SW170QT, UK; ^3^Department of Respiratory Therapy, Manipal College of Allied Health Sciences, Manipal Academy of Higher Education, Manipal 576104, Karnataka, India; ^4^Department of Infectious Diseases, Kasturba Medical College, Manipal Academy of Higher Education, Manipal 576104, Karnataka, India

## Abstract

**Background:**

There is ambiguity in the literature regarding hypoalbuminemia as a cause of extravascular lung water and acute respiratory distress syndrome (ARDS) outcomes. The aim of the study was to determine if low serum albumin on admission leads to lung deaeration and higher lung ultrasound score (LUSS) in ARDS patients. *Patients and Methods*. It was a prospective observational study in which 110 ARDS patients aged between 18 and 70 years were recruited. Serum albumin level and lung ultrasound score were assessed on the day of ICU admission. Length of ICU stay and hospital mortality were recorded.

**Results:**

The mean and standard deviation of serum albumin level in mild, moderate, and severe ARDS was 2.92 ± 0.65 g/dL, 2.91 ± 0.77 g/dL, and 3.21 ± 0.85 g/dL, respectively. Albumin level was not correlated to the global LUSS (Pearson correlation *r* −0.006, *p*=0.949) and basal LUSS (*r* −0.066, *p*=0.513). The cut-off value of albumin for predicting a prolonged length of ICU stay (≥10 days) in ARDS patients was <3.25 g/dL with AUC 0.623, *p* < 0.05, sensitivity of 86.67%, specificity of 45.45%, and 95% confidence interval (CI) [0.513–0.732], and on multivariate analysis it increased the odds of prolonged ICU stay by 8.9 times (Hosmer and Lemeshow *p* value 0.810, 95% CI [2.760–28.72]). Serum albumin at admission was not a predictor of mortality. LUSS on the day of admission was not useful to predict either a prolonged length of ICU stay or mortality. Basal LUSS contributed about 56% of the global LUSS in mild and moderate ARDS, and 53% in severe ARDS.

**Conclusion:**

Serum albumin level was unrelated to LUSS on admission in ARDS patients. Albumin level <3.25 g/dL increased the chances of a prolonged length of ICU stay (≥10 days) but was not associated with an increase in mortality. LUSS on the day of admission could not predict either a prolonged length of ICU stay or mortality. This trial is registered with CTRI/2019/11/021857.

## 1. Introduction

Albumin contributes up to 80% of colloid osmotic pressure in the physiological state [1]. However, protein-induced high oncotic intravascular gradient as proposed by Starling has now been proven inaccurate, and the interstitial compartment itself has high protein concentration [2]. It has been said that an intact glycocalyx with a particular minimum concentration of plasma proteins (albumin at least >1 g/dL) ensures adequate vascular endothelial barrier function [2, 3]. Vascular barrier is disrupted not due to hypoalbuminemia, but due to the destruction of endothelial glycocalyx due to systemic inflammation and ischemic-reperfusion injury [2, 4]. These concepts question the role of albumin in altering lung air:fluid ratio, which can be accurately estimated from bedside lung ultrasound (LUS) [5].

Contrary to this, previous literature shows that extravascular lung water is increased in patients with low albumin in critically ill head injured patients, as evidenced by lung ultrasound, though neurologic pulmonary oedema could have been a confounding factor [6]. A low serum protein is a strong predictor of acute respiratory distress syndrome (ARDS) development in sepsis patients [7]. Bedside LUS has been proven to represent the extent of pulmonary ventilation; thus, we used LUS to estimate the lung aeration [8]. Since literature is replete with dissensions regarding the role of albumin in worsening lung aeration, we wanted to determine if albumin was associated with lung deaeration.

## 2. Aim

The study aimed to determine whether serum albumin is correlated to lung deaeration as evidenced by lung ultrasound score (LUSS) in ARDS patients. We also planned to determine if serum albumin and LUSS on the day of ICU admission could be valuable predictors of prolonged length of ICU stay and mortality.

## 3. Materials and Methods

### 3.1. Setting

A single centre observational study was conducted from 19th November 2019 to 23rd January 2021 on ARDS patients admitted to the ICU.

### 3.2. Inclusion and Exclusion Criteria

The enrolled population consisted of 110 ARDS patients.

#### 3.2.1. Inclusion Criteria

- ARDS patients who did not test positive for corona virus disease 2019 (non-COVID-19).
- Adult patients aged between 18 years and 70 years on either noninvasive ventilation (NIV) or invasive mechanical ventilation (IMV).

#### 3.2.2. Exclusion Criteria

- Patients with chest trauma, massive blood transfusion, and heart failure.
- Patients with obvious causes of fluid overload like acute pulmonary oedema due to acute kidney injury and acute heart failure, which may cause sudden fluctuation in serum albumin levels.
- Patients with known hypoalbuminemia states-nephrotic syndrome, chronic kidney disease, cirrhosis, burns, chronic congestive heart failure, sarcoidosis, Crohn's disease, Celiac disease, chronic liver disease with ascites, ulcerative colitis, elderly with age >70 years, and patients with malignancy.
- Patients with post-albumin transfusion, patients on dialysis, and patients with surgeries involving gastrointestinal tract.

### 3.3. Sample Size

The sample size was estimated considering the expected correlation coefficient between serum albumin and total or global LUSS score as at least 0.25 and power of 80%, alpha error of 5%, and one-sided hypothesis, as 93, rounded off to 100. The formula used to calculate sample size (*n*) was

(1) $$\begin{array}{c} n=\frac{{\left(Z_{1-\beta }+Z_{\left(1-\alpha \right)/2}\right)}^{2}}{r^{2}/\left(1-r^{2}\right)}, \end{array}$$

where *r* is the correlation coefficient, *Z*_1−*α*/2_ is the desired confidence level, and 1 − *β* is the power.

### 3.4. Ethics

Institutional Ethics Committee approval was obtained (IEC project number 591/2019) and Clinical Trial Registry-India (CTRI) registration was done prior to recruitment of the first study participant (CTRI/2019/11/021857). Informed consent was obtained from legally authorized representative of the patient prior to recruitment for the study.

### 3.5. Methodology

**Figure 1:**
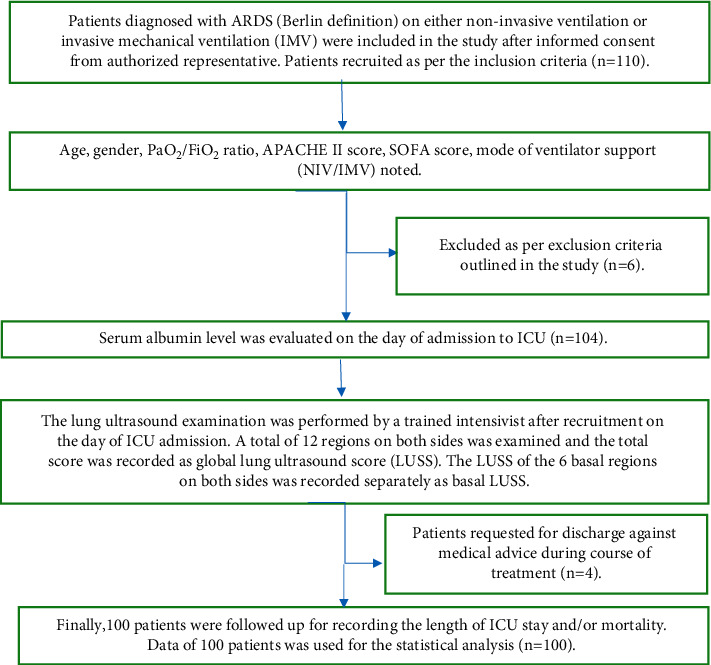
Flowchart depicting the methodology of the study.

The procedure involved in the study is explained (Figure 1).

LUS examination was done by a trained intensivist as early as possible after sending serum to estimate albumin level. Six regions in each lung were examined for LUSS. The investigator calculating the LUSS was kept unaware of the serum albumin value. Each hemithorax was divided into anterior (between sternal border and anterior axillary line), lateral (between anterior axillary line and posterior axillary line), and posterior (from posterior axillary line to as far posteriorly as the probe can be placed in the hemithorax in a patient with slight tilt) [9]. Each hemithorax was further divided into equal halves as upper or superior region and basal or inferior region [9]. So a total of 12 lung regions were scanned in both hemithoraxes and total or global LUSS was recorded from 0 to 36. The LUSS in the inferior regions of both hemithoraxes was referred to as basal LUSS. The scoring for the lung ultrasound appearance was as follows (Table 1) [9].

The length of ICU stay was considered prolonged if it was ≥10 days [10]. We intended to calculate the cut-off value of serum albumin and global LUSS on the day of ICU admission as per receiver operating characteristic (ROC) curve analysis which would predict a prolonged length of ICU stay of ≥10 days and mortality. We also intended to perform univariate and then multivariable logistic regression analysis for the variables age, gender, Acute Physiology and Chronic Health Evaluation (APACHE II score), Sequential Organ Failure Assessment (SOFA) score, the value of partial pressure of oxygen to fraction of inspired oxygen (PaO2/FiO_2_), serum albumin level, and global LUSS to predict a prolonged length of ICU stay (≥10 days) and mortality.

### 3.6. Data Collection

Data of age, gender, diagnosis, APACHE II score, SOFA score, PaO_2_/FiO_2_, type of ventilator support—NIV or IMV, ventilator settings, serum albumin level, global and basal LUSS, days of ICU stay, and outcome in terms of hospital mortality were recorded. For LUS examination, Phillips machine model CX 50 (Phillips Healthcare, 3000 Minuteman Road, Andover, USA) with curvilinear probe was used. The primary outcome was serum albumin level and LUSS (global and basal) on the day of ICU admission. The secondary outcome was in terms of length of ICU stay and mortality. Supplementary material titled “Data Sheet Albumin.pdf” is added in the supplementary section, consisting of the patient data on which study's statistical analysis was done.

## 4. Statistical Analysis

Data analysis was done using statistical software IBM SPSS (Statistical Package for the Social Sciences) software (IBM Corp. Released 2012.IBM SPSS statistics for Windows, version 22.0 Armonk, NY: IBM). Correlation between serum albumin level and LUSS was done using Pearson correlation test. A positive correlation means that as one parameter value increases, the other also increases. A negative correlation means that as one parameter increases, the other decreases. Pearson's correlation *r* < 0.3 was considered a weak correlation, *r* of 0.3–0.69 was considered a moderate correlation, and *r* > 0.7 was considered a strong correlation. A *p* value <0.05 was considered statistically significant.

Whether albumin level, global LUSS, and basal LUSS were able to predict a prolonged length of ICU stay (≥10 days) was analysed by ROC curve analysis after calculating area under the curve (AUC). A cut-off value of serum albumin for predicting prolonged length of ICU stay was calculated according to ROC curve analysis, and sensitivity, specificity, positive predictive value, negative predictive value, and diagnostic accuracy were calculated. One-way analysis of variance (ANOVA) was used to compare means of two or more samples.

Multivariable logistic regression analysis was done for APACHE II score, SOFA score, PaO_2_/FiO_2,_ serum albumin level, and global LUSS to predict prolonged length of ICU stay and survival. The logistic regression model development variables were based on the significance in the univariate analysis of the seven variables—age, gender, APACHE II score, SOFA score, PaO_2_/FiO_2_, serum albumin, and global LUSS—to predict a prolonged length of ICU stay and mortality. Those with a *p* value <0.25 in the univariate analysis were included in the multivariable analysis, except the variables serum albumin and global LUSS. It has been validated in literature that variables with a *p* value <0.25 in univariate analysis may be included in the multivariable logistic regression [11]. As our primary research question involved the variables serum albumin and global LUSS, they were included in the multivariable analysis irrespective of the *p* value in the univariate analysis. Thus, we considered low serum albumin according to the cut-off value as per our ROC analysis in the logistic regression model to predict the prolonged length of ICU stay and survival. Hosmer and Lemeshow goodness of fit test for logistic regression was done. Hosmer and Lemeshow test *p* value was calculated to ensure the model is a good fit (*p* > 0.05). The percentage contribution of basal LUSS to global LUSS in all three categories of ARDS was calculated.

## 5. Results

A total of 110 ARDS patients were included in the study. After applying the exclusion criteria, six patients were excluded from the study. Another four patients requested discharge to another hospital during the course of treatment; thus, the length of ICU stay and outcome could not be recorded. Finally, data of 100 patients was used for the analysis. The demographic characteristics and mean and standard deviation (SD) of the variables are depicted (Table 2).

Pulmonary cause of ARDS (pneumonia, drowning) was present in 43 cases whereas extrapulmonary cause of ARDS (acute febrile illness, sepsis, poisoning, acute pancreatitis, and melioidosis) was present in the remaining 57 patients.

The mean and SD of global LUSS, basal LUSS, and albumin and the percentage contribution of the basal LUSS to the global LUSS in the three categories of ARDS are depicted (Table 3).

The basal LUSS contributed to about 56% of the global LUSS in even the mild and moderate ARDS patients and 53% in severe ARDS patients.

The APACHE II scores of the patients with mild, moderate, and severe ARDS are as depicted (Table 4).

There was a significant difference between APACHE II scores of mild and severe ARDS, with *p* < 0.05.

In our analysis, there was no correlation between serum albumin and global LUSS (Pearson correlation coefficient *r* −0.006, *p* 0.949) and between serum albumin and basal LUSS (Pearson correlation coefficient *r* −0.066, *p* 0.513).

**Figure 2:**
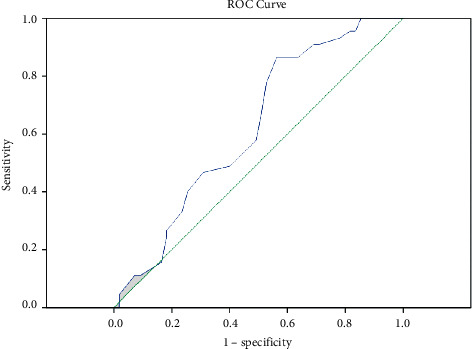
ROC curve having AUC 0.623 with a cut-off value of serum albumin (<3.25 g/dL) to predict a prolonged length of ICU stay (≥10 days).

The cut-off value of albumin from ROC curve as per AUC for predicting a longer length of ICU stay (≥10 days) in ARDS patients was <3.25 g/dL, and it was significant (AUC 0.623, *p* < 0.05, CI [0.513–0.732], sensitivity 86.67%, specificity 45.45%, positive predictive value 56.52%, negative predictive value 80.65%, and diagnostic accuracy 64%). There were 69 patients out of 100 who had serum albumin <3.25 g/dL. Out of those 69 patients, 39 (56%) had a prolonged length of ICU stay, whereas only 6 out of 31 (19%) patients with albumin >3.25 g/dL had a prolonged length of ICU stay. The ROC curve for serum albumin and a prolonged length of stay is depicted (Figure 2).

From our study, we did not have a significant cut-off value for predicting mortality from baseline serum albumin value (AUC 0.486, *p*=0.823, CI [0.359–0.614]). Similarly, global LUSS on the day of admission was not able to predict a significant longer length of ICU stay (AUC 0.554, *p*=0.353, CI [0.441–0.667]) or mortality (AUC 0.605, *p*=0.09, CI [0.492–0.717]). There was a significant moderate negative correlation between global LUSS and PaO_2_/FiO_2_ (Pearson correlation *r* −0.520, *p* < 0.05) and between basal LUSS and PaO_2_/FiO_2_ (Pearson correlation *r* −0.445, *p* < 0.05), indicating that LUSS was correlated to oxygenation. Univariate analysis showed that APACHE II score (*p*=0.047), SOFA score (*p*=0.062), PaO_2_/FiO_2_ ratio (*p*=0.042), and serum albumin (*p*=0.020) were significant predictors of prolonged length of stay in ICU (*p* < 0.25). Even though global LUSS (*p*=0.292) was not significant after the univariate analysis to predict prolonged length of ICU stay, it was included in the multivariable logistic regression as it was a variable included as a part of the primary research question.

Univariate analysis of the variables predicting outcome in terms of mortality showed that age (*p*=0.103), APACHE II score (*p* < 0.001), SOFA score (*p*=0.003), PaO_2_/FiO_2_ (*p*=0.001), and global LUSS (*p*=0.047) were significant (*p* < 0.25). However, even though albumin (*p*=0.549) was not a significant variable after univariate analysis to predict mortality, it was included in the multivariable logistic regression as it was a variable in the primary research question.

In our study, using multivariable logistic regression analysis, the prolonged length of stay in ICU was attributed to serum albumin and PaO_2_/FiO_2_, whereas APACHE II score and PaO_2_/FiO_2_ were the factors affecting outcome in terms of survival. Albumin <3.25 g/dL increased the odds of prolonged length of ICU stay (≥10 days) by 8.9 times (95% CI 2.760–28.72, Hosmer and Lemeshow *p* value = 0.810) (Table 5).

Every unit mmHg increase in PaO_2_/FiO_2_ decreased the risk of prolonged ICU stay by 1%. Regarding survival, every unit increase in APACHE II score also decreased the chances of survival by 17.31% and every unit increase in PaO_2_/FiO_2_ increased the chances of survival by 1.014 times (Table 6). The Hosmer and Lemeshow test depicted that the model was a good fit.

## 6. Discussion

Hypoproteinemia (<5.9 g/dL) with a hypoalbuminemia level <2.4 g/dL had been concluded to be a marker of increased pulmonary permeability in sepsis and ARDS patients [12]. In a study, the pulmonary leak index was said to have decreased with the increase in serum proteins. However, the study had a small sample size of 24 patients, with 16 having ARDS and the rest having sepsis [12]. An in-depth analysis of the results proved that decreased colloid osmotic pressure with a normal permeability of vascular endothelium does not contribute to oedema. Instead, it was the increased permeability due to damaged endothelium which lead to hypoproteinemia [12]. Another study revealed that albumin permeability, as assessed by radio nucleotide technique in septic shock in animals, was increased in abdominal organs, rather than in lungs [13]. Thus, increased systemic permeability due to low albumin could not be equated to similar pulmonary vascular permeability. Trials have shown that albumin administration does not alter ICU length of stay or mortality in patients [14, 15].

However, at the other end of the spectrum, a study on 455 ICU patients with sepsis showed that hypoproteinemia predicts ARDS development and is related to poor respiratory outcome and mortality [7]. To the best of our knowledge, study on albumin level and lung deaeration in real time by objectively scoring all twelve lung regions in ARDS patients has not been done. Presently, LUS has become sacrosanct to determine lung aeration and air-fluid ratio [16]. LUSS accurately depicting lung aeration was concluded by a significant correlation between global and basal LUSS and PaO_2_/FiO_2_ after our analysis. In our study, we did not find the serum albumin level to correlate with the LUSS. The mean serum albumin was in fact higher in the severe ARDS group (3.21 g/dL) than that in the mild and moderate ARDS groups (2.92 g/dL and 2.91 g/dL, resp.). The findings may be explained by the fact that, apart from increased pulmonary vascular permeability and endothelial dysfunction, albumin levels may be affected by multiple factors like nutritional status, fever, gut losses via protein enteropathy, any damage to glomerular basement membrane, and even insulin and growth hormone levels [17]. The findings of our study were similar to the study on 600 trauma patients, where albumin <3 g/dL on the day of admission did not predict days of mechanical ventilation or ICU stay [18]. Our findings were in contrast to those of the study done on head injury patients, where an albumin level <3.5 g/dL was related to more extravascular lung water [19]. The findings could have been due to the fact that traumatic brain injury also causes endothelial dysfunction in the systemic microcirculation through arginase-1–dependent uncoupling of endothelial nitric oxide synthase [19]. This cause of endothelial dysfunction, along with contributory neurogenic pulmonary oedema, could have influenced the findings of that study. The conclusion by Polito and Martin that there is no evidence that albumin resuscitation prevents development of respiratory dysfunction validates that hypoalbuminemia is unrelated to higher LUSS in ARDS, as we found in our study [20].

Even though albumin is an integral part of the endothelial surface layer, it has been shown that the endothelial surface functions effectively with even minimal albumin [2, 3]. The vascular barrier function is maintained by intact glycocalyx and is probably the reason behind the findings of the study [2]. It has also been shown in another study that even if albumin leaks intravascularly into the alveolus, it neither increases surface tension nor affects surfactant function [21]. All these findings validate the lack of correlation between albumin and higher LUSS, as we found. Thus, deaeration observed in LUS in ARDS patients appear to be unrelated to serum albumin levels. It may be due to significant neutrophil migration to alveoli along with chemokines and cytokines, as is evident by the fact that neutrophils may consist of 50% of the cells in bronchoalveolar lavage in ARDS patients [22].

The finding in our study that albumin <3.25 g/dL causes a prolonged length of ICU stay, defined by ≥10 days, may be due to various other beneficial systemic effects of albumin such as antioxidant properties, free radical scavenging, and inhibition of platelet aggregation [2, 10]. Low albumin level also causes altered protein binding of antimicrobials, especially in sepsis patients, with increased volume of distribution [23]. It may lead to inadequate treatment [23]. Thus, it is the systemic rather than the pulmonary endothelial effects of albumin which could have contributed to prolonged ICU stay in patients with low albumin in our study. This finding was similar to the previous studies in sepsis patients [7, 24]. However, after multivariable analysis, low albumin at admission could not predict survival in our study. This was contrary to the findings of the study on elderly sepsis patients aged >65 years, where univariate analysis showed 3.1 g/dL in survivors and 2.6 g/dL in nonsurvivors [24]. Our results could have been different because all the patients in our study were ≤70 years. Another retrospective study on 88 ARDS patients concluded that albumin >3.5 mg/dL had higher chances of survival than <2.9 mg/dL, regardless of septic shock [24]. Nevertheless, the similarity with our results was that, in their study as well, they found APACHE II to be a better predictor of mortality on admission than albumin level [25].

We found the mean global LUSS as 24.02 ± 4.01. This was similar to the findings of a study on LUS predicting clinical severity and outcome, where a mean global LUSS score of 22.1 ± 4.9 was found in the ARDS group [8]. However, the mean global LUSS in the mild and moderate ARDS group in our study was much higher (20.58 and 24.53, resp., versus 9.9 ± 1.7, 14.0 ± 1.4) as compared to the previous study [8]. The correlation of LUSS to PaO_2_/FiO_2_ as we found in our study also corroborated with the study described above [8]. However, we cannot predict a cut-off LUSS for determining a prolonged ICU stay or survival unlike the above mentioned study, which concluded that LUSS was 24.3 ± 3.8 in the death group, compared to 12.7 ± 2.9 in the survival group [8]. The LUSS on admission was statistically nonsignificant in our study to predict mortality. The change in LUSS over period of stay in the ICU could be used in future studies to predict mortality. A study on Radiologic Severity Index (RSI) scores using chest X-ray and computerized tomography scans of lung in patients with lower respiratory tract infections had a similar conclusion [26]. The authors had concluded that though a baseline RSI could not predict 30-day mortality, the change in RSI predicted mortality [26].

A study on ARDS patients concluded that posterior LUSS was a significant contributor to the global LUSS and was the only score that increased significantly with ARDS severity [27]. Similarly, we evaluated the six basal lung regions separately and found that the basal LUSS was the predominant part of the global LUSS in all three categories of ARDS (about 56%), even as early as on the day of admission to ICU. This could be explained by the model of asymmetric fractal branching of both airways and vasculature in the lungs [28]. The asymmetrical branching leads to air flow difference of 1.46 : 1 with subsequent branching, which is aggravated in lung injury [29]. This, coupled with the cranial displacement of diaphragm in supine position and compression of basal alveoli due to pressure of abdominal contents, may accentuate the disturbance in ventilation-perfusion in the basal lung areas [28]. Our finding of the more significant contribution of basal regions to the global LUSS on admission even in mild and moderate ARDS may justify prone positioning even in these groups of patients. Early awake prone positioning in COVID-19 patients leads to significant improvement in oxygenation and avoidance of intubation [29]. Since our study proved that basal regions are more involved even in mild and moderate ARDS patients, early proning might help.

Multivariable analysis done in our study showed that, on the day of admission, every unit increase in APACHE II score and reduction in PaO_2_/FiO_2_ decrease the chances of survival by 17.31% and 1.104%, respectively. The findings of our study are similar to those of a study on predictors of mortality in 94 ARDS patients on admission, where it was shown that only APACHE II was a reliable predictor [30]. APACHE III score has been shown to predict mortality in ICU patients, but controversy has been reported in predicting outcome for ARDS patients [31]. We had a higher mortality in the pulmonary cause of ARDS group (46.5%) compared to the extrapulmonary group (22.8%), which could have also influenced survival outcome in our study. However, serum albumin level was not associated with mortality in our study. The lack of benefit of albumin administration in liberation from mechanical ventilation and mortality has been reported in the FADE trial [32]. A systematic review with sequential analysis of all-cause mortality from major trials like SAFE, ALBIOS, and EARSS concluded that human albumin solutions did not reduce all-cause mortality in sepsis of any severity, even in patients with hypoalbuminemia [12, 13, 33, 34]. There were a number of limitations of the study. It was a single-centre study. Serum albumin and LUS examination was done only once on the day of admission to ICU. We did not have equal number of patients in mild, moderate, and severe ARDS for a better comparison of the basal region contribution to the total LUSS between the three categories of ARDS. For assessment of the outcomes of ARDS patients in terms of mortality and length of ICU stay, organ dysfunction developing later during ICU stay could have also affected outcomes. However, we performed multivariable logistic regression analysis for APACHE II score, SOFA score, PaO_2_/FiO_2,_ serum albumin level, and global LUSS on admission to ICU to predict length of ICU stay and survival. Our findings are limited to ARDS patients without causes of fluid overload like oliguric renal failure and cardiogenic pulmonary oedema and thus cannot be generalised to all patients of ARDS.

## 7. Conclusion

On the day of admission to ICU, serum albumin is unrelated to the degree of lung deaeration as depicted by LUSS in ARDS patients. Serum albumin level <3.25 g/dL increased the chances of a prolonged length of ICU stay (≥10 days) but was unable to predict mortality. LUSS on the day of admission to the ICU was unable to predict either a prolonged length of ICU stay or mortality.

## Figures and Tables

**Table 1:** Lung ultrasound score calculation based on ultrasound findings.

Point for each lung zone	Degree of lung aeration	Pattern
0 points	Normal	A lines
1 point	Moderate loss	Well separated B lines
2 points	Severe loss	Coalescent B lines
3 points	Complete loss	Lung consolidation

**Table 2:** Depiction of demographic and variables of the study.

Variables	Baseline character (*N* = 100) Mean ± SD
Age (years)	51.23 ± 12.44
Gender	
Male (%)	63
Female (%)	37
APACHE II score	16.26 ± 6.16
SOFA score	9.44 ± 4.4
PaO_2_/FiO_2_ ratio	155.5 ± 54.60
Global LUSS	24.02 ± 4.01
Basal LUSS	13.31 ± 2.15
Serum albumin (g/dL)	2.97 ± 0.76
Basal LUSS as a percentage of global LUSS	55.73 ± 5.15
Mild ARDS	24
Moderate ARDS	58
Severe ARDS	18

**Table 3:** Depiction of the LUSS and albumin level in the three categories of ARDS.

Variables	ARDS category							
	Mild (*N* = 24)		Moderate (*N* = 58)		Severe (*N* = 18)		Total	
	Mean	SD	Mean	SD	Mean	SD	Mean	SD
Global LUSS Basal LUSS	20.58 11.46	4.77 2.41	24.53 13.76	2.93 1.57	26.94 14.33	2.71 2.03	24.02 13.31	4.01 2.15
Basal LUSS as percentage of global LUSS	56.17%	5.21%	56.36%	5.14%	53.11%	4.55%	55.73%	5.15%
Serum albumin (g/dL)	2.92	0.65	2.91	0.77	3.21	0.85	2.97	0.76

**Table 4:** Depiction of the mean and SD of the APACHE II scores in the three categories of ARDS (*p* < 0.05 between mild and severe ARDS, one-way ANOVA on post hoc test).

ARDS category	APACHE II score		
	Number of patients	Mean	SD
Mild	24	14.63	5.74
Moderate	58	15.97	6.06
Severe	18	19.39	6.22
Total	100	16.26	6.16

**Table 5:** Depiction of the multivariable logistic regression of the variables in the study to predict prolonged length of ICU stay.

Variables	*B*	S.E.	Wald	Degrees of freedom	*p* value	OR	95% CI for OR	
							Lower	Upper
APACHE II score	−0.058	0.049	1.438	1	0.230	0.943	0.858	1.038
SOFA score	−0.117	0.069	2.855	1	0.091	0.889	0.777	1.019
PaO_2_/FiO_2_ ratio	−0.012	0.005	4.992	1	<0.05	0.988	0.977	0.998
Global LUSS	0.017	0.071	0.060	1	0.806	1.017	0.886	1.168
Serum albumin <3.25 g/dL	2.186	0.598	13.386	1	<0.05	8.903	2.760	28.722

**Table 6:** Depiction of the multivariable logistic regression of the variables in the study to predict survival.

Variables	*B*	S.E.	Wald	Degrees of freedom	*p* value	OR	95% CI for OR	
							Lower	Upper
Age	0.004	0.024	0.025	1	0.875	1.004	0.957	1.053
APACHE II score	−0.191	0.061	9.871	1	<0.05	0.8269	0.734	0.931
SOFA score	−0.048	0.069	0.484	1	0.486	0.953	0.831	1.092
PaO_2_/FiO_2_ ratio	0.014	0.006	5.035	1	<0.05	1.014	1.002	1.026
Global LUSS	0.014	0.084	0.029	1	0.864	1.015	0.860	1.197
Serum albumin <3.25 g/dL	−0.028	0.340	0.007	1	0.934	0.972	0.500	1.892

## Data Availability

The data which have been used in the statistical analysis of this study are given in supplementary information files titled “Data Sheet Albumin.pdf.”

## Abbreviations

ARDS: Acute respiratory distress syndrome
APACHE II: Acute Physiology and Chronic Health Evaluation
SOFA: Sequential Organ Failure Assessment
ICU: Intensive care unit
LUS: Lung ultrasound
LUSS: Lung ultrasound score
CI: Confidence interval
OR: Odds ratio
*B*: Exponentiation of the B coefficient
SE: Standard error.

## Additional Points

*Highlights of the Study*. (1) Low serum albumin does not lead to lung deaeration in ARDS patients as evidenced by real time ultrasound. (2) Low serum albumin at admission can predict a prolonged length of stay in intensive care unit yet does not predict mortality. (3) Basal lung ultrasound score is the predominant contributor to global lung ultrasound score, not only in moderate-severe ARDS, but also in mild ARDS. (4) Global lung ultrasound score in ARDS at admission does not predict prolonged length of stay or mortality. (5) APACHE II score and oxygenation on the day of admission to intensive care unit predict mortality in ARDS patients. The lung ultrasound examination was done by the investigators without any additional expenditure for the patients.

## Supplementary Materials

Supplementary Materials Data sheet comprising of age, gender, APACHE II score, SOFA score, ARDS category—mild, moderate, or severe—serum albumin on the day of admission to ICU, global and basal lung ultrasound scores, oxygenation in terms of PaO_2_/FiO_2_ ratio, length of ICU stay, and outcome in terms of survival is provided as “Data Sheet Albumin.pdf” in the supplementary materials file. The manuscript has been written according to Strengthening the Reporting of Observational Studies in Epidemiology (STROBE) guidelines, which is available as “STROBE_checklist_cross-sectional.docx.” in the supplementary materials file.
