# Supplementary material for: Correlation of Serum Albumin Level to Lung Ultrasound Score and Its Role as Predictors of Outcome in Acute Respiratory Distress Syndrome Patients: A Prospective Observational Study
Source: Crit Care Res Pract. 2021 Dec 7;2021:4594790. doi: 10.1155/2021/4594790 (PMC8670905; doi:10.1155/2021/4594790)
Supplement: Supplementary Materials — Data sheet comprising of age, gender, APACHE II score, SOFA score, ARDS category—mild, moderate, or severe—serum albumin on the day of admission to ICU, global and basal lung ultrasound scores, oxygenation in terms of PaO2/FiO2 ratio, length of ICU stay, and outcome in terms of survival is provided as “Data Sheet Albumin.pdf” in the supplementary materials file. The manuscript has been written according to Strengthening the Reporting of Observational Studies in Epidemiology (STROBE) guidelines, which is available as “STROBE_checklist_cross-sectional.docx.” in the supplementary materials file. [file 4594790.f1.zip › 4594790.f1/Data Sheet Albumin.pdf]

sno	Hospital Numb	Age	Gender	APACHE II score	SOFA score
1	2559693	58	M	20	16
2	3456028	46	M	11	10
3	3454771	69	M	10	5
4	3454102	50	M	8	7
5	3431339	56	M	28	18
7	3446458	50	M	16	16
8	3440311	25	M	15	10
9	3399489	36	F	17	10
10	2982448	65	M	21	7
11	3312648	50	F	4	4
12	3351944	56	M	16	3
13	3424366	70	F	22	8
14	3414704	45	M	15	14
15	3400999	32	F	4	3
16	3174036	71	M	12	4
17	3403649	36	M	19	15
18	3403025	49	F	19	12
19	3400851	28	F	10	6
20	3404095	65	F	15	13
21	3398810	32	F	20	15
22	3409293	34	M	26	10
23	3408273	36	M	3	9
24	3420496	30	F	12	3
25	3417505	59	M	7	6
26	3405313	55	M	29	10
27	3406219	68	M	25	11
28	3404517	48	F	18	11
30	3399701	51	M	6	6
31	3400440	57	M	19	17
32	3400457	38	F	12	12
33	3398327	70	M	22	9
34	3398350	40	F	2	2
35	3428059	62	M	5	5
36	3432284	53	F	18	10
37	3432373	55	M	16	11
38	3463620	64	M	19	14
39	3461200	70	M	19	15
40	3463395	58	F	14	6
41	3454865	59	F	24	16
42	3464730	43	M	14	15
43	2367252	63	F	25	11
44	3465319	45	M	15	12
46	3464252	37	M	14	7
47	3465987	54	M	19	19
48	3458206	62	M	22	11

49	3466221	50	F	22	14
50	3297288	61	M	23	6
51	3488681	27	M	7	4
52	3488733	38	F	12	9
53	3487108	49	M	22	10
54	3467924	37	F	19	9
55	3468136	60	M	14	10
56	3410927	48	M	13	7
57	3465705	49	M	14	3
61	3485302	54	F	9	5
62	3261441	60	M	14	3
63	3457674	54	M	10	9
64	3483211	68	F	14	6
66	2570479	38	M	16	9
67	2686002	56	M	20	7
68	3469488	44	F	21	9
69	2960456	59	F	23	10
70	3481538	40	M	10	6
72	2694948	65	F	11	5
73	3455744	65	F	12	2
74	3482444	47	F	15	5
75	3480481	47	M	12	8
76	3476597	47	M	23	10
77	3476168	40	M	19	20
78	3476554	43	F	11	13
79	2958161	55	M	23	13
80	2667286	64	M	22	12
81	3473815	60	F	23	12
82	3474474	45	F	17	16
84	3475124	36	F	18	15
85	3475127	60	M	16	6
86	3477736	37	M	10	6
88	3478512	63	M	27	15
89	3479355	42	F	12	12
90	3461774	43	M	20	3
91	3425781	68	M	23	7
92	3469101	69	M	13	13
93	3473935	65	F	14	10
94	3479159	58	M	19	10
95	3349464	56	M	13	6
96	3485313	23	F	9	3
97	3465128	42	F	15	4
98	3486675	41	M	20	12
99	3479939	50	M	28	20
100	3488409	23	M	3	3
101	3226933	64	M	23	12
102	3490590	56	M	12	9

103	3489559	65	M	18	10
104	3489836	55	M	22	12
105	3490572	50	M	11	6
106	3487839	66	M	22	15
107	3487828	54	M	17	2
108	3488454	70	F	14	7
109	3487839	66	F	23	11
110	2919727	31	F	26	9

ARDS category - Mil	PaO_2/FiO_2 ratio	LUSS(Global)	LUSS (Basal)	LUSS(Basal) score as a p
1	290	23	13	57
2	180	23	12	52
1	201	16	10	63
1	248	25	13	52
2	191	25	16	64
1	238	18	12	67
2	146	30	15	50
3	87	33	17	51
3	90	30	15	50
3	59	28	14	50
2	160	25	14	56
2	183	23	14	61
2	150	25	13	52
1	260	15	9	60
2	133	24	12	50
1	240	32	16	50
2	185	24	12	50
1	220	19	11	58
3	80	24	12	50
1	214	24	12	50
1	271	16	9	56
2	120	28	16	57
2	145	29	15	52
2	125	27	13	48
3	83	26	11	42
2	146	21	11	52
2	142	30	16	53
2	116	20	14	70
2	123	24	12	50
1	253	24	12	50
2	195	25	14	56
1	207	28	16	57
2	147	25	14	56
3	79	26	14	54
2	160	27	14	52
1	255	22	14	64
3	90	28	17	61
2	137	21	15	71
3	68	27	16	59
2	158	26	14	54
2	150	17	9	53
3	89	24	13	54
1	223	11	6	55
2	117	28	16	57
2	120	22	12	55

2	130	27	15	56
2	137	25	13	52
2	106	24	14	58
2	132	24	13	54
3	85	24	14	58
1	240	20	12	60
1	209	17	9	53
2	195	25	13	52
3	100	24	12	50
2	190	24	14	59
2	118	21	13	62
1	226	18	11	61
2	150	26	15	58
2	164	20	12	60
1	217	24	13	54
1	211	27	14	52
2	137	28	16	57
1	230	17	10	59
2	177	25	14	56
1	228	17	8	47
2	104	24	12	50
2	167	21	12	57
2	134	23	13	57
2	189	24	13	54
2	155	30	17	57
2	190	26	15	58
2	120	26	16	62
2	112	24	13	54
2	145	24	12	50
2	130	24	15	63
2	140	22	14	64
2	132	26	14	54
2	144	24	14	58
2	144	23	12	52
2	160	23	13	57
3	77	30	17	57
2	193	22	13	59
1	211	22	13	59
1	237	21	12	57
2	138	28	16	57
2	171	25	15	60
1	215	21	10	48
2	136	28	16	57
3	92	29	16	55
2	191	19	13	68
2	105	30	16	53
2	112	26	14	54

3	86	26	14	54
2	104	25	13	52
3	86	27	14	52
3	79	23	11	48
1	203	17	10	59
2	161	18	12	67
3	66	26	14	54
3	65	30	17	57

Serum Albumin	Outcome of ICU stay	Death	Length of ICU stay (Days)
2.5	survived	0	18
2.3	survived	0	20
2.3	survived	0	13
3.4	survived	0	8
2.3	survived	0	9
2.3	survived	0	10
1.8	survived	0	4
3	survived	0	18
3.1	EXPIRED	1	25
4.1	survived	0	4
2.1	EXPIRED	1	26
2.2	EXPIRED	1	5
2.9	survived	0	10
3.6	survived	0	4
2.9	survived	0	17
2.9	survived	0	4
2.5	survived	0	4
2.2	survived	0	4
3.4	survived	0	13
2.7	survived	0	8
4.4	survived	0	8
3.1	survived	0	12
3.8	survived	0	13
2.5	survived	0	11
3.6	survived	0	10
2.8	survived	0	6
2.5	survived	0	10
2.1	survived	0	7
2.7	EXPIRED	1	6
3.1	survived	0	15
2	EXPIRED	1	7
2.9	survived	0	8
3	survived	0	13
3	survived	0	12
2.6	survived	0	14
2.8	EXPIRED	1	5
1.7	EXPIRED	1	17
2.7	survived	0	16
3.2	EXPIRED	1	2
2.5	EXPIRED	1	5
4.7	EXPIRED	1	4
3.8	EXPIRED	1	1
2.6	survived	0	6
3.1	EXPIRED	1	1
2.7	survived	0	12

1.8	survived	0	17
2.9	EXPIRED	1	10
4	survived	0	11
2.7	EXPIRED	1	16
2.7	EXPIRED	1	2
2.9	survived	0	9
2.9	survived	0	4
3.9	survived	0	6
3.1	EXPIRED	1	15
1.8	survived	0	5
4.5	survived	0	4
3.1	survived	0	10
3.3	survived	0	5
3.2	EXPIRED	1	11
3.8	survived	0	7
2.8	survived	0	4
3.3	EXPIRED	1	2
3.2	survived	0	28
3	survived	0	10
3.4	survived	0	3
3.2	EXPIRED	1	13
3.4	survived	0	5
4.6	EXPIRED	1	2
3.6	EXPIRED	1	6
2.4	survived	0	20
2.1	EXPIRED	1	3
4	survived	0	7
2.8	EXPIRED	1	8
3.2	survived	0	7
1.9	EXPIRED	1	5
2.6	survived	0	15
2.6	survived	0	40
1.8	EXPIRED	1	15
2.2	survived	0	10
1.9	survived	0	13
3.6	EXPIRED	1	4
3.3	survived	0	4
3	survived	0	6
1	survived	0	9
2.3	survived	0	18
2.9	survived	0	11
2.9	EXPIRED	1	3
4.6	EXPIRED	1	7
5.2	EXPIRED	1	2
3.6	survived	0	3
4.5	EXPIRED	1	2
3.2	Survived	0	12

2.8	Survived	0	9
2.5	Survived	0	8
4	Survived	0	12
2.8	EXPIRED	1	10
3.4	Survived	0	10
3.5	Survived	0	7
1.4	EXPIRED	1	10
3.3	EXPIRED	1	4
